# Modeling Tissue- and Mutation- Specific Electrophysiological Effects in the Long QT Syndrome: Role of the Purkinje Fiber

**DOI:** 10.1371/journal.pone.0097720

**Published:** 2014-06-03

**Authors:** Vivek Iyer, Kevin J. Sampson, Robert S. Kass

**Affiliations:** Department of Pharmacology, College of Physicians and Surgeons of Columbia University, New York, New York, United States of America; Baylor College of Medicine, United States of America

## Abstract

Congenital long QT syndrome is a heritable family of arrhythmias caused by mutations in 13 genes encoding ion channel complex proteins. Mounting evidence has implicated the Purkinje fiber network in the genesis of ventricular arrhythmias. In this study, we explore the hypothesis that long QT mutations can demonstrate different phenotypes depending on the tissue type of expression. Using computational models of the human ventricular myocyte and the Purkinje fiber cell, the biophysical alteration in channel function in LQT1, LQT2, LQT3, and LQT7 are modeled. We identified that the plateau potential was important in LQT1 and LQT2, in which mutation led to minimal action potential prolongation in Purkinje fiber cells. The phenotype of LQT3 mutation was dependent on the biophysical alteration induced as well as tissue type. The canonical ΔKPQ mutation causes severe action potential prolongation in both tissue types. For LQT3 mutation F1473C, characterized by shifted channel availability, a more severe phenotype was seen in Purkinje fiber cells with action potential prolongation and early afterdepolarizations. The LQT3 mutation S1904L demonstrated striking effects on action potential duration restitution and more severe action potential prolongation in Purkinje fiber cells at higher heart rates. Voltage clamp simulations highlight the mechanism of effect of these mutations in different tissue types, and impact of drug therapy is explored. We conclude that arrhythmia formation in long QT syndrome may depend not only on the basis of mutation and biophysical alteration, but also upon tissue of expression. The Purkinje fiber network may represent an important therapeutic target in the management of patients with heritable channelopathies.

## Introduction

Congenital long QT syndrome (LQTS) is a heritable family of arrhythmias caused by mutations in 13 genes encoding ion channel complex proteins[1]. These mutations exert their effect through altered ion channel gating and/or trafficking, resulting in prolongation of action potential duration (APD). This prolongation manifests as lengthening of the QT interval on the electrocardiogram, providing an electrophysiological substrate for arrhythmia formation. While the majority of the characterization of arrhythmias in this disorder to date has involved the ventricular myocyte (VM), mounting evidence implicates the cardiac specialized conduction system, comprised of the Purkinje fiber (PF) network, as a particularly important trigger of arrhythmias[2]–[6]. PF cells (PFC) have distinctive action potential (AP) characteristics, including the property of automaticity, longer APD, and lower plateau membrane potential[7]. Each of these features may uniquely predispose these cells to arrhythmia formation.

Mathematical modeling has been employed to simulate genetic and acquired channelopathies and assay their effects on cardiac tissue[8], [9]. We have recently developed comprehensive integrative models of the electrophysiology of both the human VM[10] and PFC[11]. By virtue of their biophysically detailed Markov representations of ion channels (constrained to data obtained from human ion channels in heterologous expression), these models are well-suited to the study of LQT mutations, through targeted modification of the processes responsible for channel dysfunction.

In this study, we create models of LQTS in VM and PFC to investigate the hypothesis that LQTS-related mutations may manifest their phenotype in a tissue-specific manner. Several disease-causing mutations are simulated in each cardiac tissue type. We demonstrate that arrhythmia formation in LQTS may depend not only upon the specific mutation and biophysical alteration but also upon the tissue of expression, supporting an important role for the PFC.

## Results

### LQT1 and LQT2 mutations have a mild phenotype in PFC

**Figure 1:**
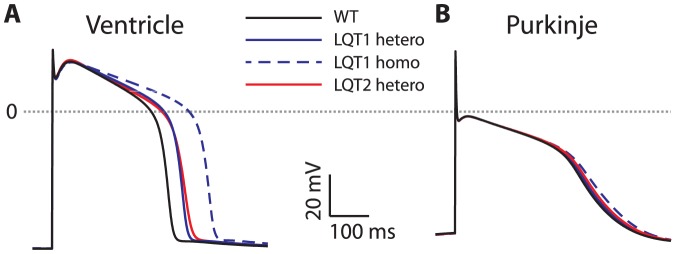
Differential response of VM (panel A) and PF (panel B) to mutation in IKs and IKr channels, leading to LQT1 and LQT2, respectively. Compared to wild type (WT, black trace), heterozygous LQT1 (blue trace) and LQT2 (red trace), modeled as a 50% reduction in whole cell conductance, VM APs are prolonged, while PF are mildly affected. Homozygous LQT1 (100% reduction in IKs conductance, dashed blue trace) leads to substantial APD prolongation in VM, and trivial further prolongation in PF.

As LQT1 and LQT2 mutations often result in complete loss-of-function of the affected KCNQ1 or HERG allele, respectively (through defective trafficking or biophysical changes in channel function)[1], idealized heterozygous mutations were simulated by reducing peak conductance of IKs or IKr by 50% to simulate heterozygous LQT1 or LQT2, respectively. Figure 1 shows the resulting APs obtained at 1 Hz stimulation frequency. Heterozygous LQT1 mutation prolonged APD by only 8 ms in PFC, in contrast to VM, in which a 36 ms APD prolongation is seen. Mutation in LQT2 prolongs PFC APD by 28 ms and VM APD by 46 ms. Clinically, homozygous LQT1 mutation leads to the Jervell-Lange-Nielsen syndrome[1], which is characterized by severe QT prolongation. Homozygous LQT1, simulated as IKs deletion, results in further prolongation of VM APD (408 ms versus 304 ms WT), which is in stark contrast to the PFC APD which exhibits minimal prolongation of 15 ms.

The differential degree of APD prolongation in PFCs compared to VMs is only partially explained by differences in baseline IKr and IKs current density. IKr shows a 60% lower whole cell peak conductance in PFC, but IKs whole cell peak conductance is 60% greater. The lower plateau potential in PFC plays a large role in the tissue-specific response via less complete activation of IK channels. IKs channel isochronal 1000 ms open probability at the PFC plateau potential measures 0.22, compared with 0.82 at the VM plateau potential.

### Different potential mechanisms of arrhythmia formation in Andersen-Tawil Syndrome

The Andersen-Tawil Syndrome (ATS, or LQTS type 7[12]) is linked to heterozygous loss of function in Kir2.1, which encodes the inward rectifier current IK1, through defective channel trafficking[13]. Previous work demonstrates that with dominant negative suppression of IK1 current, delayed afterdepolarizations (DADs) are observed in simulated VM[14], and that a PFC focus may be responsible for this arrhythmia[15].

**Figure 2:**
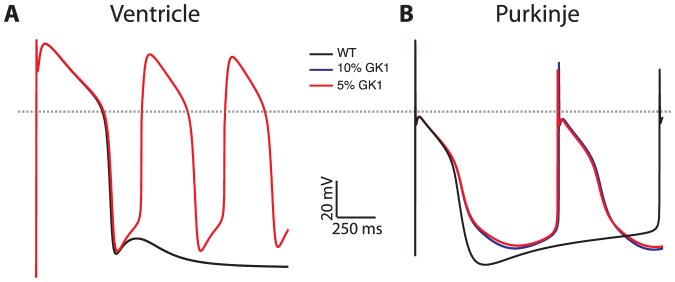
Arrhythmias in Andersen-Tawil syndrome, modeled as dominant negative suppression of IK1 current, have different mechanisms in VM (panel A) versus PF (panel B) myocytes. For VM, compared to WT (black trace) reduction of IK1 conductance by 90% (blue) leads to a large delayed afterdepolarization. Further reduction to 95% (red) increases the size of the DAD, triggering repetitive action potentials. For PF, reduction of IK1 conductance maximum diastolic potential is less polarized, leading to increased automaticity. Unstimulated PF APs show basic cycle length of automaticity of 2250 msec, which decreases to 1260 with 90% IK1 reduction.

In Figure 2, dominant negative IK1 suppression is simulated in both VM and PFC. Arrhythmias occur via different mechanisms in the two cell types. In the VM model, progressive downregulation of IK1 leads to DADs of increasing amplitude occurring immediately after the AP (panel A), until another AP is triggered leading to repetitive membrane depolarization. The mechanism is identical to that observed in guinea pig VM[14]. Analogous downregulation of IK1 in PFC shows no DADs (panel B). The slow diastolic depolarization underlying PFC automaticity reaches threshold for AP formation earlier than in VM, with a less polarized maximum diastolic potential. The result is an approximate doubling of the intrinsic firing rate stemming from unopposed HCN current. Our simulations show that VM carrying ATS mutations are capable of triggered activity as an arrhythmic mechanism, while corresponding PFC show no DADs but display enhanced automaticity, another potential mechanism for arrhythmia.

### Effect of the SCN5A sodium channel mutations on PFC and VM

SCN5A inactivation defects underlie AP prolongation in LQT3 via gain of function, producing a persistent component of INa termed late current, or INaL. These defects can occur as a result of alteration of several distinct biophysical processes. Using several different mutations in SCN5A, we explored whether these alterations may manifest in a tissue- and/or mutation-specific manner.

The first studied and most thoroughly characterized mutant, ΔKPQ, results in channels that enter a ‘bursting’ mode of gating: channel entry into this mode of gating is rare, but recovery from this mode is infrequent, trapping the channel in a state in which it cannot inactivate[8]. This results in time-invariant persistent channel opening, which generates inward late current that prolongs APD. Given the lower plateau potential in PFC, and consequent larger driving force for INa entry, our initial hypothesis was that a more pronounced effect of ΔKPQ would be seen on the PFC APD compared with VM.

**Figure 3:**
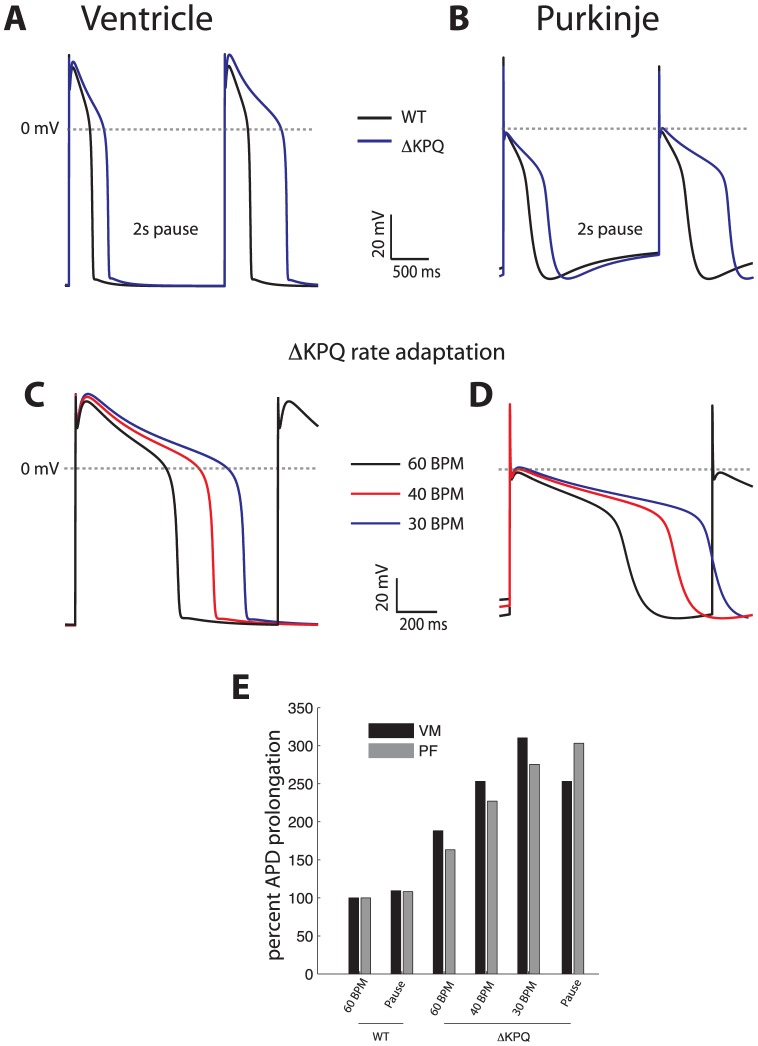
VM and PFC carrying the SCN5A ΔKPQ mutation exhibit similarly severe APD prolongation. Panel A and B) Effect of a 2 second pause for VM and PFC, respectively, compared with 1000 msec (last beat of drive train shown for WT, black trace, and mutant cells, blue trace). Panels C and D) Steady state rate adaptation for VM and PFC, respectively (30 BPM: blue trace, 40 BPM: red trace, 60 BPM: black trace). E) Summary results for APD prolongation for each cell type for different stimulus protocols; VM: black bars, PF: gray bars.

**Figure 4:**
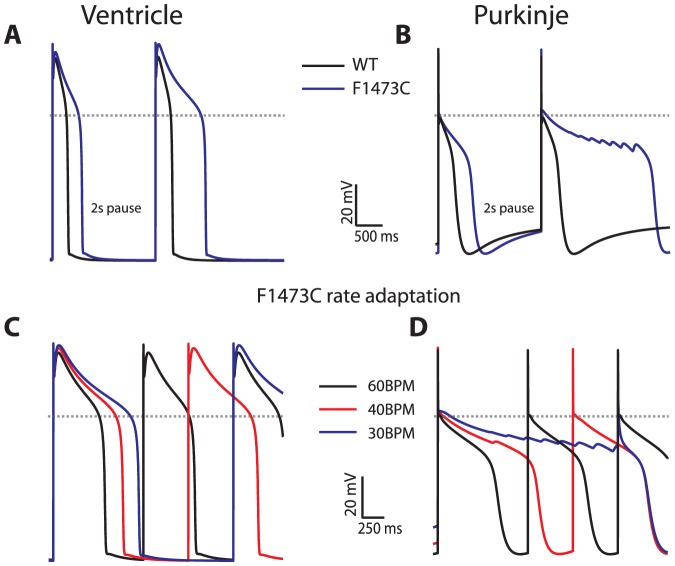
Differential effect on APD for VM versus PF of the SCN5A F1473C mutation. Panels A and B) Effect of a 2 second pause for VM and PFC, respectively, compared with 1000 msec (last beat of drive train shown for WT, black trace, and mutant cells, blue trace). APD in the F1473C shows pause-dependent increase, with spontaneous oscillations in membrane potential from sequential early afterdepolarizations seen post-pause for PF but not VM. Panels C and D) Rate adaptation for VM and PFC, respectively (30 BPM: blue trace, 40 BPM: red trace, 60 BPM: black trace. In VM cells, substantial APD prolongation is seen (on the order of that seen with ΔKPQ mutation) with pronounced bradycardia dependence. In PF APs, severe APD prolongation is seen at 60 BPM, for 40 BPM stable alternans are seen (with a small early afterdepolarization on every other beat), and at 30 BPM the membrane fails to repolarize.

Figure 3 shows the resulting APs using a rate constant for the bursting transition that produces 0.5% persistent current, corresponding to previous studies[8], [16]. At 1 Hz, APD is prolonged by 69% and 61% in VM and PFC, respectively. After a 2 second pause, VM APD carrying the mutation is prolonged to 866 ms (increase of 185%), compared to PFC at 892 ms (increase of 146%). Similarly, more severe APD prolongation is noted in both tissue types during bradycardia (pacing at 30 BPM and 40 BPM, panels C and D), as summarized in Figure 4E.

The finding that VM are more affected by ΔKPQ than PFC was surprising, since in PFC there is a larger driving force for sodium entry during the plateau. We hypothesized that the more depolarized diastolic potential in PFC may force a larger fraction of SCN5A channels into unavailable states, preventing entry into the pathologic bursting states.

Therefore, we next tested the role of channel availability by simulating the LQT3 SCN5A mutation F1473C, a mutation characterized clinically by severe QT interval prolongation and heavy arrhythmia burden[17], and characterized biophysically by a rightward shift in the availability curve of 10 mV and large amplitude late current. F1473C channels were simulated with the same bursting rate as ΔKPQ, along with a shift in the availability curve of 10 mV in the depolarized direction (see expanded methods for values of rate constants)[18]. Figure 4 shows APs at 1 Hz, demonstrating marked APD prolongation (598 ms in VM versus 677 ms in PFC). As with ΔKPQ, following a 2 sec pause, APD is prolonged for both tissue types. Strikingly, for PFC following a pause, repetitive early afterdepolarization (EAD) formation is observed as a result of severe APD prolongation. Similar findings are observed for continuous pacing at slower rates. For PFC at 40 BPM, there is a single EAD in every other beat (AP alternans, seen in panel D), while at 30 BPM APs with an EAD alternate with shorter APs. These findings clearly establish an arrhythmogenic substrate within PFC for the F1473C mutation.

### S1904L mutation in SCN5A confers a more severe phenotype in PFC than VM through a different channel gating mechanism

In contrast to typical SCN5A LQT3 mutations, for which the phenotype is exacerbated by bradycardia and rest, the SCN5A S1904L LQTS mutation was described in a young patient with only a mildly prolonged QT interval who developed palpitations with mild exercise[9]. We have recently characterized these mutant channels, which exhibit late current as a result of late channel reopenings without a bursting component.

**Figure 5:**
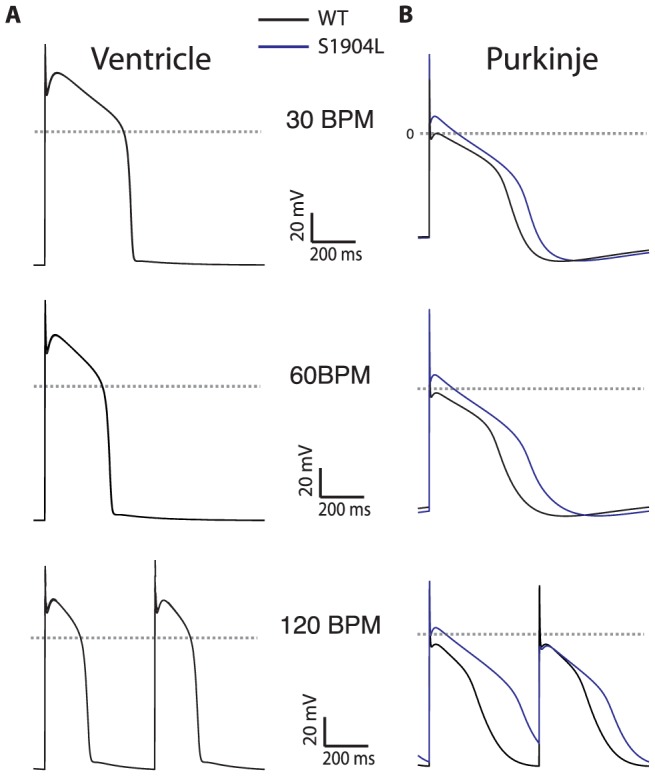
The S1904L mutation, characterized clinically by arrhythmia at low levels of exertion, simulated at clinically relevant pacing frequencies. The mutation exhibits minimal response in VM (panel A, traces overlap) and greater APD prolongation in PF (panel B), with APD alternans seen at 120 BPM (lower row). WT cells: black trace, S1904L: blue trace.

This channel behavior is recapitulated by reducing the inactivation rate constant and reducing the rate constant of entry into deeper buried inactivated states by a factor of 5 and 6, respectively, generating 0.28% late current (as seen experimentally[9]). Figure 5A shows the predicted consequence of the S1904L in the VM model. As previously shown at 1 Hz[9], the impact on VM is minimal (no prolongation). In contrast, PFC bearing this mutation exhibit 120 ms APD prolongation at 1 Hz. More rapid pacing (120 BPM) exacerbates this phenotype, prolonging PFC APD by 200 ms but still with minimal change in VM. The result is a markedly flattened restitution curve for the PFC model even with a mild increase in rate. A predominant rate-dependent effect on PFC explains the emergence of the clinical phenotype with exercise as well as the propensity for arrhythmia formation despite minimal QT interval prolongation.

### Voltage clamp simulations reveal mechanism of mutation-specific differences in LQT3

**Figure 6:**
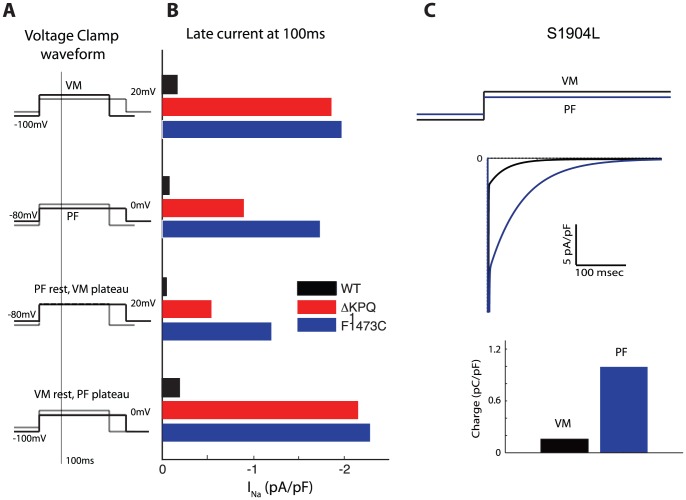
Voltage clamp protocols demonstrate themechanism of late current generation in LQT3 mutations. Panel A illustrates the voltage clamp waveform used. Panel Bdemonstrates the important role of resting membrane potential and plateaupotential on degree of late sodium current in ΔKPQ (red bar)and F1473C mutations (blue bar) compared to WT (black bar).For a “VM-like” voltage clamp protocol (holding −100 mV,step potential 20 mV, top row), similar persistent current isseen for KPQ and F1473C mutations. When holding potential ischanged to −80 mV mimicking PF AP resting potential, second row,persistent current is less for the KPQ mutation than for the F1473C mutation (whichhas increased availability at this potential). Third andfourth rows show the individual effect of altering plateau and resting potentials.Panel C) Response of step potentials simulating VM (black)and PF (blue) in cells carrying the S1904L mutation.The “PF-like” voltage clamp waveform produces more inwardcurrent at all time points, leading to more accumulated inward charge as demonstratedbelow in the bar graph.

The differential response of the PFC AP to ΔKPQ and F1473C suggests that the shift in channel availability (proarrhythmic) may counteract the effect of hyperpolarized diastolic potential in PFC (antiarrhythmic). To directly test the contributions of these factors individually, voltage clamp simulations were performed at different holding potentials (−100 mV and −80 mV, corresponding to the diastolic potential of VM and PFC, respectively) with steps to different test potentials (+20 and 0 were selected, corresponding to plateau potentials). Absolute late sodium current at 100 ms was calculated, alongside percent persistent current (normalized to peak) for WT, ΔKPQ, and F1473C. At a holding potential of −100 mV, both ΔKPQ and F1473C show similar amounts of late current, regardless of step potential (and driving force), in contrast to WT cells (first two sets in Figure 6A). At a holding potential of −80 mV, F1473C exhibits a far greater late current than ΔKPQ. Lesser late current is produced by ΔKPQ at depolarized resting potentials due to gating of channels into unavailable states (top row of states in Figure 1B of Clancy[19]) from which they cannot burst nor conduct current during the plateau of the AP. Thus, the higher driving force for current entry is counterbalanced by lower fractional occupancy of bursting states that carry INaL. When availability is shifted in F1473C channels, direct comparison of the -100 to +20 mV simulation (mimicking a VM AP profile) and −80 to 0 mV simulation (mimicking a PFC AP profile) shows more absolute late current in the PFC-like profile. This substantial increase in late current underlies the more severe prolongation of APD in PFCs for the F1473C mutation.

We also used the square voltage clamp protocol for the S1904L mutation to mimic VM and PFC AP morphologies. In the “VM-like” voltage clamp, the magnitude of the late current (and late current fraction) is small compared to the PFC-like voltage clamp profile (see Figure 6B). The prolonged time course of inactivation at 0 mV compared to +20 mV (time constant of current decay 67 ms at 0 mV versus 30 ms at +20 mV) confers a larger functional impact in the context of the S1904L mutation, allowing persistent channel opening during the step potential. This corresponds to 85% smaller net summed current entry (net charge) during the test pulse for a VM-like profile (bar graph, Figure 6B). Repetition of these voltage clamp waveforms at 2 Hz does not appreciably alter net current entry, suggesting that the larger effect at higher frequencies in PFC is due to smaller counterbalancing net repolarizing current such as the transient outward current.

## Discussion

The major findings of this study are that a) LQT1 and LQT2 exert a relatively mild phenotype in PFC; b) different mechanisms of arrhythmia formation are seen in Andersen-Tawil syndrome based on cardiac tissue type; c) the canonical LQT3 mutant ΔKPQ has severe phenotype in both VM and PFC tissue types; d) in contrast to ΔKPQ, the LQT3 mutant F1473C shows a particularly severe phenotype in PFC with repetitive membrane depolarizations due to shifted availability of sodium channels; and e) the LQT3 mutant S1904L exerts a mild phenotype, but is more severe in PFC with strong rate-dependent exacerbation of phenotype. Numerous lines of evidence implicate the PF system as important in the genesis of LQTS-related arrhythmias, including findings from electrophysiological studies of affected patients. Our findings, which stem from inherent differences in the electrophysiology of the PFC compared to the VM, provide a theoretical basis with which to understand these clinical observations.

### Arrhythmias in the ATS may result from enhanced PFC automaticity

The mechanism of arrhythmia formation in ATS has not been established. Previous simulation studies showed that in VM with dominant negative suppression of IK1, DADs were observed[14], [20]. Our findings in VM reproduce this behavior, with repetitive membrane depolarization upon critical IK1 reduction (Figure 3). PFC with dominant negative IK1 suppression, however, did not show evidence of DADs. Instead, we saw a graded increase in intrinsic automaticity. This suggests that abnormal automaticity (stemming from the cardiac conduction system) and/or triggered activity (from VM) may underlie the ventricular arrhythmias seen in ATS. The Sanders lab has reported sharp “Purkinje potentials” preceding ventricular premature beats during electrophysiology studies in ATS patients[15]. The bidirectional ventricular tachycardia often seen in these patients may reflect alternation between two different PFC foci with enhanced automaticity, via a mechanism similar to that described by Weiss[21]. Our results thus provide a plausible mechanism that may underlie the clinical demonstration of His-Purkinje system involvement.

### The importance of the plateau potential in determining APD for LQT1 and LQT2

In all mutations studied, one common theme is the importance of the plateau potential (and plateau current magnitudes) in determining the effect of a mutation. Our results simulating LQT1 and LQT2 (Figure 1) show a more profound APD-prolonging effect of IK deletion in VM. It has been widely reported that baseline IK density is different in VM and PFC[22], and as such, the dependence on IK for membrane repolarization (and consequence of deletion of these currents) would be expected to be different in each cell type. However, we found that differences in channel activation may underlie the greater APD prolongation in VM. Indeed, at the VM plateau potential, our results predict a nearly four-fold greater IKs channel open probability. Previous experiments using APs as command waveforms predicted a similar result, wherein larger IK current was elicited using an AP with ventricular morphology[23]. Taken together, these results suggest that not only channel density, but AP shape regulates the effect of LQT1 and LQT2 mutations in different tissue types.

Our model for LQT1 was relatively simple, in that a simple change in conductance was simulated rather than a more subtle loss-of-function resulting from altered kinetics. Pourrier and Nattel reported that accessory subunits of the IKs channel may express differentially in different cardiac tissue types, with a higher density in Purkinje fibers[24]. A disproportionate reduction in PFC IKs via differential expression of subunits may modulate the predictions of the simpler model described herein.

### Altered Na+ channel availability and defects in inactivation modulate tissue-specific effects of LQT3 mutations

Clancy[8] showed that AP prolongation with the ΔKPQ deletion in SCN5A can arise from sodium channels that enter an abnormal bursting mode, in which the channel is trapped in a configuration from which it cannot transition to inactivate.

We initially hypothesized that for PFC carrying ΔKPQ, a larger driving force for sodium current entry during the relatively hyperpolarized plateau provides the basis for more severe APD prolongation. We did not find this to be the case, instead observing a slightly more pronounced APD prolongation in VM compared to PFC. The steady-state availability curve for SCN5A predicts 0.68 fractional availability at the PFC diastolic potential of −83 mV and 0.85 fractional availability at the corresponding VM potential of −91 mV. The additional fraction of inactivated channels in PFC cannot transition into the bursting states to produce persistent current.

Our previous work described the LQT3 mutation F1473C, characterized by severe QT prolongation that is further exacerbated by bradycardia and substantial late sodium current[18]. When implemented into the PFC model, the results indicate that the shift in availability seen with F1473C compensates for the relatively depolarized resting potential seen in this tissue type. With the consequent greater availability of mutant channels, the enhanced driving force for sodium current entry in PFC causes disproportionate prolongation of APD. Moreover, we demonstrated in this study that PFC are prone to EADs, the putative cellular triggers of ventricular arrhythmia, and stable AP alternans promoted by slow pacing rates. These findings suggest that disruption of PFC electrophysiology was responsible for the affected patient's unusually heavy burden of ventricular arrhythmia clinically, which required defibrillator implantation as a neonate[17] and which improved markedly upon faster pacing[25].

Our finding that channel steady-state availability impacts channel function in a tissue-specific manner led us to explore whether our results have important implications for drug therapy. We tested two different local anesthetic drugs, flecainide (modeled as a charged local anesthetic SCN5A blocker) and lidocaine (modeled as an uncharged local anesthetic SCN5A blocker which affects channel availability) on our PFC model of F1473C. In the drug free state, severe APD prolongation is seen along with multiple EADs, particularly in response to a 2 second pause. Flecainide was modeled as a 20% block in sodium current and lidocaine as a 20% block in sodium current plus a leftward shift of the availability curve of SCN5A. We identified that with lidocaine, the availability shift of lidocaine conferred greater efficacy in terms of APD shortening and abolishing EADs (unlike flecainide). These simulations raise the intriguing possibility that drug efficacy may vary in a tissue-specific manner in LQTS patients.

The LQT3 mutation S1904L is unique in that APD prolongation and ventricular arrhythmias occur with faster heart rates (unlike in ΔKPQ or F1473C). Furthermore, arrhythmias occurred with mild exercise in the proband carrying this mutation[9]. Our previous characterization of this mutation showed minimal effect on ventricular APD at physiological pacing frequencies (although an effect was measurable at >200BPM[9]). In our current study, we show that VM exhibit minimal change in AP duration at both 60BPM and 120BPM, whereas for PFC a more pronounced effect is seen. Via this mechanism, a heterogeneous repolarization substrate emerges preferentially at higher heart rates, explaining the propensity toward arrhythmia formation during exercise.

### Impact of concomitant repolarizing current blockade on LQTS phenotype

Given its relatively slow activation, IK in cardiac cells is thought to play a larger role under conditions that cause AP prolongation. Acquired LQTS, as occurs via partial blockade of IKr associated with QT-prolonging drug therapy, may reflect unmasking of a subclinical channelopathy, through deletion of an important repolarizing influence that in WT cells may be redundant. As studied by Gintant in canine Purkinje fibers[26], AP prolongation from repolarizing current blockade may manifest prominently in PF. Since there are known difficulties in estimating the values of IK density in VM, and this density may vary from cell to cell, we explored whether variation in IK in cells carrying LQT3 mutations may affect our findings. As shown in Figure S3 in File S1, we found that concomitant IK block leads to substantial AP prolongation in VM carrying LQT3 mutations (both ΔKPQ and F1473C), but no EADs are seen even with IK block up to 50%. This suggests that while repolarizing currents play a larger role in the presence of coincident gain-of-function LQTS mutations, it is the degree of late current augmentation that determines whether triggered activity occurs.

We furthermore performed simulations in another model of the human ventricular action potential, and with both epicardial and endocardial profiles, to establish that our findings in LQT3 generalize across tissue layer and do not reflect artifactual behavior unique to one ionic model of the ventricular myocyte. For these simulations (described in further detail in File S1), the model of Ten Tusscher[27] was utilized. The findings confirm those obtained using the model described here (Figure S4 in File S1).

### Nature of the interaction between PFC and VM impacts arrhythmogenesis at the organ level and potential therapeutic strategies

The cell type-specific responses to different LQTS mutations theoretically provide a substrate for arrhythmogenesis, by increasing the heterogeneity of repolarization (contributing to dispersion of APD and dispersion of the QT interval). Evidence is accumulating that the PF network is organized in complex sheets adjacent to the endocardial surface of the heart that only interact with VM at sparse PFC-VM junctions. This geometry may facilitate propagation of EADs that occur preferentially and focally in PFC to neighboring cell layers, suggesting the potential for reentry and proarrhythmia.

Antiarrhythmic therapy is the standard of care in the management of many patients with LQTS, with an adjunctive role of implantable cardioverter-defibrillators to reduce mortality associated with sudden cardiac death. A central role of the PFC and specialized conduction system may suggest specific pharmacologic targets that may be tailored to a specific mutation. Furthermore, since PFC can be targeted anatomically, alternate modes of therapy, for example, via catheter ablation may be possible. We have recently characterized a PFC-specific mutation in the transient outward current, identified in patients with idiopathic ventricular fibrillation who were successfully treated with catheter ablation[28]. Since the His-Purkinje system inserts endocardially, PF triggers in other heritable channelopathies may evolve to represent attractive targets of ablation therapy, as has recently been reported in a canine model of LQT3[29] and during electrophysiology studies of patients with LQTS[2].

### Limitations

A first limitation of the simulations shown is that conclusions apply to the single cell, but without multicellular analysis, its impact on the whole organ level remains untested. Future work by our group will explore the potential for triggering of arrhythmias from these cellular proarrhythmic states.

A second limitation lies in the formulation of calcium handling utilized. This is a weakness of every current model of the PFC. We plan to update our model to incorporate what is known about PFC ultrastructure and more accurately reflect intracellular calcium cycling. Such an addition may further elucidate the link between altered membrane repolarization and triggers of arrhythmia.

### Conclusions

The Purkinje fiber network has long been implicated as a source of ventricular arrhythmia. We show that for several mutations linked to LQTS, a different phenotype emerges in different cardiac tissue types, often predicting a more severe phenotype in PFC. Our findings suggest the potential for a new type of regulation of heritable channelopathies in which not only channel dysfunction but the variable impact on different cardiac tissues can modulate the severity of phenotype, with important implications for therapy of these conditions.

## Methods

The whole-cell mathematical models utilized in this study have been described previously[10], [11]. Briefly, these models are composed of systems of coupled nonlinear differential equations representing channel occupancies, which are integrated numerically using a fourth order Runge-Kutta ordinary differential equations solver with an adaptive time-step. Computations were performed in C++ language on a 4-processor workstation, allowing single cell computations in real time. Further details regarding the computational models can be found in previous work[10], [11].

The previously developed PFC model[11] is implemented without change. In order to model the mutations identically in each tissue type, the Markov representations of membrane currents from the VM model were replaced with the corresponding models from the PFC, specifically the slow component of the delayed rectifier potassium current (IKs) and the fast sodium current INa[11]. To ensure appropriate balance of densities of the fast component of the delayed rectifier IK (IKr) and IKs after this change, conductance of these currents was adjusted to produce appropriate response of APD to specific blockers of each component (E-4031 for IKr[30] and chromanol 293B for IKs[31]). Appropriate APD for human epicardial VM was reproduced, chiefly through a 20% increase in the conductance of the L-type calcium current (matching recent data recording L-type current density in human right[32] and left[33]–[35] VM). A complete description of model parameters is provided in the expanded methods section in the File S1. The model reproduces key features of human epicardial VM, including appropriate rate dependent shortening of APD, as seen in Figure S1 in File S1. In Figure S2 in File S1 the updated model output is compared to a recorded action potential and the original model of Iyer et al [10], showing minimal difference in action potential shape and duration.

Source code for the two utilized models is provided in the supplementary material online (Source Code S1).

## Supporting Information

File S1 **This file contains Figure S1–Figure S4, Table S1, and Table S2.** Figure S1, Action potentials for VM (panel A) and PF (panel B) at 0.5 Hz (dash-dot), 1 Hz (dashed), and 2 Hz (solid) stimulation frequency. Features unique to PF APs include lower plateau potential, prolonged APD, and slow diastolic depolarization (automaticity). Figure S2, Panel A) Simulated original Iyer model[3] action potential obtained at 1 Hz pacing (black trace) versus that of the model with update described in Methods (blue trace). Panel B) Human left ventricular epicardial action potential recorded at 1 Hz from Nabauer[1] compares favorably to the model output. Figure S3, Simulated drug block of IK in cells carrying LQT3 mutations shows minimal additional AP prolongation in PF cells carrying the ΔKPQ mutation (panel A) compared to VM (panel B). WT: gray trace, ΔKPQ: solid black, ΔKPQ with 50% IKs block: gray dashed, ΔKPQ with 50% IKr block: black dashed. Panel C) For the F1473C mutation in VM, substantial concomitant block of IK (50% reduction) fails to produce EADs in the post-pause beat, which are seen during drug-free simulations of PF cells. F1473C: solid, F1473C with 50% IKs block: gray dashed, F1473C with 50% IKr block: black dashed. Figure S4, Use of an alternate model of the human ventricular action potential, that of Ten Tusscher[4], shows a similar response to LQT3 mutations. Top row: Epicardial (panel A) and endocardial (panel B) ventricular cell models are utilized to study wild type (WT, black trace), ΔKPQ (red trace) and F1473C mutant sodium channels following a 2 second pause This VM model shows no evidence of early afterdepolarization formation matching findings in Figures 3 and 4. In Panel C, bradycardia is simulated with 40 BPM pacing in an epicardial cell, also demonstrating no early afterdepolarizations, similar to findings from the modified Iyer model[3]. Panel D) The S1904L mutation simulated in the Ten Tusscher model at 2 Hz (red trace) shows superimposed action potentials over wild type (WT, black trace), similar to Figure 5. Table S1, Changes to the original human ventricular myocyte model [3]. Table S2, Parameter changes to simulate mutations.
(DOC)

Source Code S1 (ZIP)
