# Supplementary material for: Modeling Tissue- and Mutation- Specific Electrophysiological Effects in the Long QT Syndrome: Role of the Purkinje Fiber
Source: PLoS One. 2014 Jun 3;9(6):e97720. doi: 10.1371/journal.pone.0097720 (PMC4043730; doi:10.1371/journal.pone.0097720)
Supplement: File S1 — This file contains Figure S1–Figure S4, Table S1, and Table S2. Figure S1, Action potentials for VM (panel A) and PF (panel B) at 0.5 Hz (dash-dot), 1 Hz (dashed), and 2 Hz (solid) stimulation frequency. Features unique to PF APs include lower plateau potential, prolonged APD, and slow diastolic depolarization (automaticity). Figure S2, Panel A) Simulated original Iyer model[3] action potential obtained at 1 Hz pacing (black trace) versus that of the model with update described in Methods (blue trace). Panel B) Human left ventricular epicardial action potential recorded at 1 Hz from Nabauer[1] compares favorably to the model output. Figure S3, Simulated drug block of IK in cells carrying LQT3 mutations shows minimal additional AP prolongation in PF cells carrying the ΔKPQ mutation (panel A) compared to VM (panel B). WT: gray trace, ΔKPQ: solid black, ΔKPQ with 50% IKs block: gray dashed, ΔKPQ with 50% IKr block: black dashed. Panel C) For the F1473C mutation in VM, substantial concomitant block of IK (50% reduction) fails to produce EADs in the post-pause beat, which are seen during drug-free simulations of PF cells. F1473C: solid, F1473C with 50% IKs block: gray dashed, F1473C with 50% IKr block: black dashed. Figure S4, Use of an alternate model of the human ventricular action potential, that of Ten Tusscher[4], shows a similar response to LQT3 mutations. Top row: Epicardial (panel A) and endocardial (panel B) ventricular cell models are utilized to study wild type (WT, black trace), ΔKPQ (red trace) and F1473C mutant sodium channels following a 2 second pause This VM model shows no evidence of early afterdepolarization formation matching findings in Figures 3 and 4. In Panel C, bradycardia is simulated with 40 BPM pacing in an epicardial cell, also demonstrating no early afterdepolarizations, similar to findings from the modified Iyer model[3]. Panel D) The S1904L mutation simulated in the Ten Tusscher model at 2 Hz (red trace) shows superimposed action potential [file pone.0097720.s001.doc]

**Supplementary material**

Additional simulations are presented in this supplemental material to further investigate properties of ventricular myocyte (VM) and Purkinje fiber cell (PFC) electrophysiology.

The rate dependence of the VM model and PFC model are first explored, to ensure appropriate shortening at faster pacing rates. The results are shown in Supplement File Figure S1 below. In both cell types, the characteristic shape and duration of the action potential matches what is known experimentally. Next, we present the modified VM model action potential in comparison to both the original Iyer model action potential (AP) and an experimentally recorded action potential at 1 Hz from a human left ventricular myocyte. As seen in Supplement File Figure S2, the changes introduced (described above in Expanded Methods) impart minimal change to action potential duration, with any changes in profile attributable chiefly to the different formulation and different conductance of the delayed rectifier current. The resulting action potential profile also compares favorably with an experimentally recorded AP.

We next study the effect of concomitant IK blockade in our cell models. Given its relatively slow activation, IK in cardiac cells is thought to play a larger role under conditions that cause AP prolongation. Acquired LQTS, as occurs via partial blockade of IKr associated with QT-prolonging drug therapy, may reflect unmasking of a subclinical channelopathy, through deletion of an important repolarizing influence that in WT cells may be redundant. Since there are known difficulties in estimating the values of IK density in VM, and this density may vary from cell to cell, we explored whether variation in IK in cells carrying LQT3 mutations may affect our findings. As shown in Supplement File Figure S3, concomitant IK block leads to substantial AP prolongation in VM carrying LQT3 mutations (both ΔKPQ and F1473C), but no EADs are seen even with IK block up to 50%. This suggests that while repolarizing currents play a larger role in the presence of coincident gain-of-function LQTS mutations, it is the degree of late current augmentation that determines whether triggered activity occurs.

Multiple models of the human ventricular action potential have been developed. In order to both verify that our findings apply to other ionic models of the ventricular action potential, and also apply to simulated endocardial action potentials, we utilized the model of Ten Tusscher and colleagues and simulated identical protocols to those performed in Figures 3, 4, and 5. We show in Supplemental File Figure S4 that this ventricular cell model shows comparable behavior to the modified Iyer model used in the main figures, suggesting that the tissue specificity of effect does not reflect an artifact of the model used and also applies to different tissue layers within the ventricle.

**Supplement File Figure S1**

**
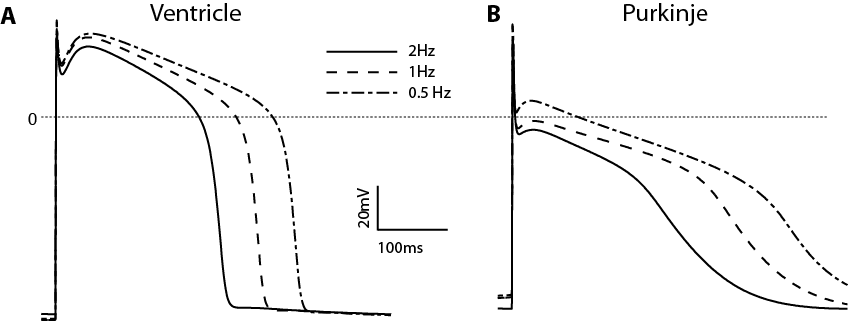
**

**Supplement File Figure S1.** Action potentials for VM (panel A) and PF (panel B) at 0.5 Hz (dash-dot), 1 Hz (dashed), and 2 Hz (solid) stimulation frequency. Features unique to PF APs include lower plateau potential, prolonged APD, and slow diastolic depolarization (automaticity).

**Supplement File Figure S2**

**Supplement File Figure S2.** Panel A) Simulated original Iyer model action potential obtained at 1 Hz pacing (black trace) versus that of the model with update described in Methods (blue trace). Panel B) Human left ventricular epicardial action potential recorded at 1 Hz from Nabauer compares favorably to the model output.

**Supplement File Figure S3**

**
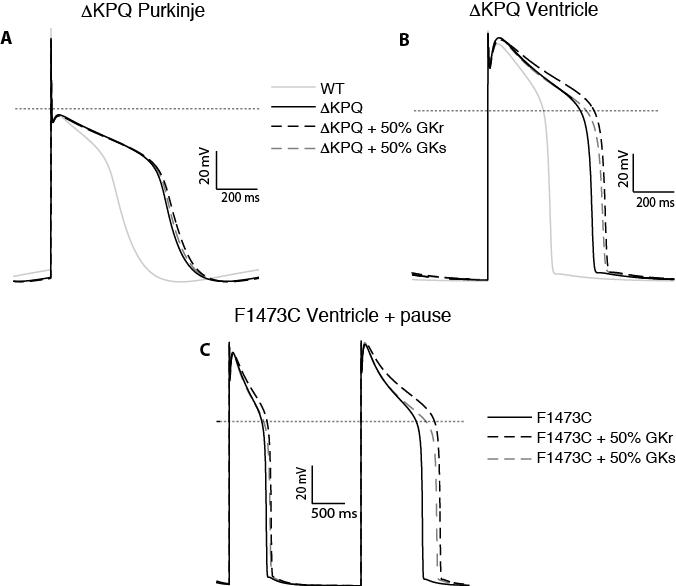
**

**Supplement File Figure S3.** Simulated drug block of IK in cells carrying LQT3 mutations shows minimal additional AP prolongation in PF cells carrying the ΔKPQ mutation (panel A) compared to VM (panel B). WT: gray trace, ΔKPQ: solid black, ΔKPQ with 50% IKs block: gray dashed, ΔKPQ with 50% IKr block: black dashed. Panel C) For the F1473C mutation in VM, substantial concomitant block of IK (50% reduction) fails to produce EADs in the post-pause beat, which are seen during drug-free simulations of PF cells. F1473C: solid, F1473C with 50% IKs block: gray dashed, F1473C with 50% IKr block: black dashed.

**Supplement File Figure S4**

**Supplement File Figure S4.** Use of an alternate model of the human ventricular action potential, that of Ten Tusscher, shows a similar response to LQT3 mutations. Top row: Epicardial (panel A) and endocardial (panel B) ventricular cell models are utilized to study wild type (WT, black trace), ΔKPQ (red trace) and F1473C mutant sodium channels following a 2 second pause This VM model shows no evidence of early afterdepolarization formation matching findings in Figures 3 and 4. In Panel C, bradycardia is simulated with 40 BPM pacing in an epicardial cell, also demonstrating no early afterdepolarizations, similar to findings from the modified Iyer model. Panel D) The S1904L mutation simulated in the Ten Tusscher model at 2 Hz (red trace) shows superimposed action potentials over wild type (WT, black trace), similar to Figure 5.

**Expanded methods**

The previously developed PFC model is implemented without change. In order to model the mutations identically in each tissue type, the Markov representations of membrane currents from the VM model were replaced with the corresponding Markov models from the PFC; specifically the kinetic models for IKs and for INa, as previously described. To ensure appropriate balance of densities of IKr and IKs after this change, conductance of these currents was adjusted to produce appropriate response of APD to specific blockers of each component (E-4031 for IKr and chromanol 293B for IKs). Appropriate APD for human epicardial ventricular myocytes was reproduced, chiefly through a 20% increase in the conductance of the L-type calcium current (matching recent data recording L-type current density in human right and left ventricular myocytes). See Supplement File Table S1 for all modifications to the original Iyer model and original parameters. Mutations were simulated as described in the main text. Complete parameter changes required to reproduce figures are provided in Supplement File Table S2. Source code is also available online.

**Supplement File Table S**1

| Parameter | Original value | New value |
| --- | --- | --- |
| GKr | 0.0186 mS/uF | 0.12 mS/uF |
| GKs | 0.0035 mS/uF | 0.02 mS/uF |
| GNa | 56.32 mS/uF | 35 mS/uF |
| GK1 | 0.125 mS/uF | 0.195 mS/uF |
| L-type P_Ca_ | 1.73 x 10^-3^ cm/sec | 2.08 x 10^-3^ cm/sec |
| L-type γ | 0.055 x [subspace Ca^2+^] | 0.037 x [subspace Ca^2+^] |
| L-type voltage dependent inactivation | 0.82 | 0.66 |

Supplement File Table S1. Changes to the original human ventricular myocyte model. Parameters are as defined in the Supplemental data of our previous work.

**Supplement File Table S**2

| Parameter | WT value | Mutation | Mutation value |
| --- | --- | --- | --- |
| INa u1 | 4.3 x 10^-8^ | ΔKPQ, F1473C | 5.0 x 10^-7^ |
| INa a3 | 3.79e-7*exp(-V/7.7) | F1473C | 3.79e-7*exp(-(V-10)/7.7) |
| INa a2 | 9.178*exp(V/29.68) | S1904L | 9.178*exp(V/29.68) / 5 |
| INa a4 | 9.178*exp(V/29.68)/100 | S1904L | 9.178*exp(V/29.68) /600 |
| GKs scale | 1 | LQT1/WT | 0.5 |
| GKs scale | 1 | LQT1/LQT1 | 0.0 |
| GKr scale | 1 | LQT2 | 0.5 |
| GK1 scale | 1 | LQT7 | 0.05 |

Supplement File Table S2. Parameter changes to simulate mutations. Parameters are as defined in the Supplemental data of our previous work.

Supplement References

1. Nabauer, M., et al., *Regional differences in current density and rate-dependent properties of the transient outward current in subepicardial and subendocardial myocytes of human left ventricle.* Circulation, 1996. **93**(1): p. 168-77.

2. Dun, W. and P.A. Boyden, *The Purkinje cell; 2008 style.* J Mol Cell Cardiol, 2008. **45**(5): p. 617-24.

3. Iyer, V., R. Mazhari, and R.L. Winslow, *A computational model of the human left-ventricular epicardial myocyte.* Biophys J, 2004. **87**(3): p. 1507-25.

4. ten Tusscher, K.H., et al., *A model for human ventricular tissue.* Am J Physiol Heart Circ Physiol, 2004. **286**(4): p. H1573-89.

5. Sampson, K.J., et al., *A computational model of Purkinje fibre single cell electrophysiology: implications for the long QT syndrome.* J Physiol, 2010. **588**(Pt 14): p. 2643-55.

6. Li, G.R., et al., *Evidence for two components of delayed rectifier K+ current in human ventricular myocytes.* Circ Res, 1996. **78**(4): p. 689-96.

7. Bosch, R.F., et al., *Effects of the chromanol 293B, a selective blocker of the slow, component of the delayed rectifier K+ current, on repolarization in human and guinea pig ventricular myocytes.* Cardiovasc Res, 1998. **38**(2): p. 441-50.

8. Li, G.R., et al., *Transmembrane ICa contributes to rate-dependent changes of action potentials in human ventricular myocytes.* Am J Physiol, 1999. **276**(1 Pt 2): p. H98-H106.

9. Beuckelmann, D.J. and E. Erdmann, *Ca(2+)-currents and intracellular [Ca2+]i-transients in single ventricular myocytes isolated from terminally failing human myocardium.* Basic Res Cardiol, 1992. **87 Suppl 1**: p. 235-43.

10. Beuckelmann, D.J., M. Nabauer, and E. Erdmann, *Intracellular calcium handling in isolated ventricular myocytes from patients with terminal heart failure.* Circulation, 1992. **85**(3): p. 1046-55.

11. Magyar, J., et al., *Effects of endothelin-1 on calcium and potassium currents in undiseased human ventricular myocytes.* Pflugers Arch, 2000. **441**(1): p. 144-9.
